# Structural Evaluation of the Spike Glycoprotein Variants on SARS-CoV-2 Transmission and Immune Evasion

**DOI:** 10.3390/ijms22147425

**Published:** 2021-07-10

**Authors:** Mohd Zulkifli Salleh, Jeremy P. Derrick, Zakuan Zainy Deris

**Affiliations:** 1 Department of Medical Microbiology & Parasitology, School of Medical Sciences, Universiti Sains Malaysia Health Campus, Kubang Kerian 16150, Malaysia; m.z.salleh@usm.my; 2 Lydia Becker Institute of Immunology and Inflammation, School of Biological Sciences, Faculty of Biology, Medicine and Health, Manchester Academic Health Science Centre, The University of Manchester, Oxford Road, Manchester M13 9PL, UK; jeremy.derrick@manchester.ac.uk

**Keywords:** SARS-CoV-2, COVID-19, spike variants, spike mutations, immune evasion, transmission

## Abstract

The emergence of severe acute respiratory syndrome coronavirus 2 (SARS-CoV-2) presents significant social, economic and political challenges worldwide. SARS-CoV-2 has caused over 3.5 million deaths since late 2019. Mutations in the spike (S) glycoprotein are of particular concern because it harbours the domain which recognises the angiotensin-converting enzyme 2 (ACE2) receptor and is the target for neutralising antibodies. Mutations in the S protein may induce alterations in the surface spike structures, changing the conformational B-cell epitopes and leading to a potential reduction in vaccine efficacy. Here, we summarise how the more important variants of SARS-CoV-2, which include cluster 5, lineages B.1.1.7 (Alpha variant), B.1.351 (Beta), P.1 (B.1.1.28/Gamma), B.1.427/B.1.429 (Epsilon), B.1.526 (Iota) and B.1.617.2 (Delta) confer mutations in their respective spike proteins which enhance viral fitness by improving binding affinity to the ACE2 receptor and lead to an increase in infectivity and transmission. We further discuss how these spike protein mutations provide resistance against immune responses, either acquired naturally or induced by vaccination. This information will be valuable in guiding the development of vaccines and other therapeutics for protection against the ongoing coronavirus disease 2019 (COVID-19) pandemic.

## 1. Introduction

More than 168 million people have been infected worldwide with coronavirus disease 2019 (COVID-19), resulting in over 3.5 million deaths (as of May 2021). COVID-19 was first detected as an atypical form of pneumonia in Wuhan, China [1]: symptoms of the disease include fever or chills, cough, difficulty breathing, headache, sore throat, loss of taste or smell, nausea and diarrhoea [2]. Globally the COVID-19 pandemic has caused substantial social, economic and political impacts. COVID-19 is caused by severe acute respiratory syndrome coronavirus 2 (SARS-CoV-2), a novel positive-strand single-stranded RNA virus which is categorised in the subfamily *Coronavirinae* of the family *Coronaviridae* and the order *Nidovirales*. Severe acute respiratory syndrome coronavirus (SARS-CoV) and Middle East respiratory syndrome coronavirus (MERS-CoV) are two pathogenic, highly transmissible viruses that belong to the same family. They all have zoonotic origins and are likely to have originated in bats [3]. It is thought that SARS-CoV and MERS-CoV were transmitted directly to humans from market civets and camels, respectively, as intermediate hosts [4]. There is much debate on how SARS-CoV-2 came into contact with humans, whether directly from bats or indirectly via an intermediate animal host, such as Malayan pangolins [5]. There is evidence that SARS-CoV-2 is similar to a coronavirus found in horseshoe bats (*Rhinolophus*), with a sequence similarity of 98.7% to the partial RNA-dependent RNA polymerase (*RdRp*) gene of the bat coronavirus strain BtCoV/4991 and 87.9% similarity to bat coronavirus strains bat-SL-CoVZC45 and bat-SL-CoVZXC21 [6].

## 2. SARS-CoV-2 Structures

Like other coronaviruses, SARS-CoV-2 is a positive-strand single-stranded RNA virus (+ssRNA), with a linear segment of RNA of approximately 30,000 bases, larger than any other RNA virus. SARS-CoV-2 virions, which are 60–140 nm in diameter, have four structural proteins: the spike (S), envelope (E), membrane (M) and nucleocapsid (N) proteins and 16 non-structural proteins (Nsp1–16) [6,7,8,9]. The nucleocapsid protein wraps around the RNA genome, encapsulated within an envelope associated with the S, E and M proteins [10]. Nsp1 facilitates RNA replication and processing and is also involved in mRNA degradation. Nsp2 mediates modulation of the host cell survival signalling pathway [11,12]. Nsp3 is a papain-like proteinase and degrades the translated polyprotein into distinct proteins [13]. Nsp4 is a membrane-spanning protein and is proposed to anchor the viral replication-transcription complex to modified endoplasmic reticulum (ER) membranes of the host cell [14]. The 3C-like proteinase Nsp5, on the other hand, is involved in viral polyprotein processing during replication [15]. Nsp6 is predicted to form a transmembrane protein and plays a vital role in the initial induction of autophagosomes from the host ER. Nsp7 and Nsp8 are RNA-dependent RNA polymerases and form a hexadecameric super-complex [16]. Nsp9 is a single-stranded RNA-binding protein and participates in viral replication [17]. Nsp10 contains two zinc-binding motifs and is crucial for the viral mRNA cap methylation [18]. The RNA-dependent RNA polymerase Nsp12 is important in the replication and transcription of the viral genome and forms a core polymerase complex with Nsp7 and Nsp8 [19]. The helicase Nsp13 binds ATP and participates in viral genome replication and transcription [7]. Nsp14 has dual activities: guanosine N7-methyltransferase activity and exoribonuclease activity; it is critical for the replication of SARS-CoV-2 [20]. The endoribonuclease Nsp15 cleaves the 5′-polyuridine motif of negative-sense viral RNA [21,22]. Nsp16 is a 2′-O-ribose-methyltransferase and methylates the ribose of the first transcribed nucleotide with S-adenosylmethionine [23]. To date, the function of Nsp11 is unknown.

## 3. Spike Glycoprotein

**Figure 1:**
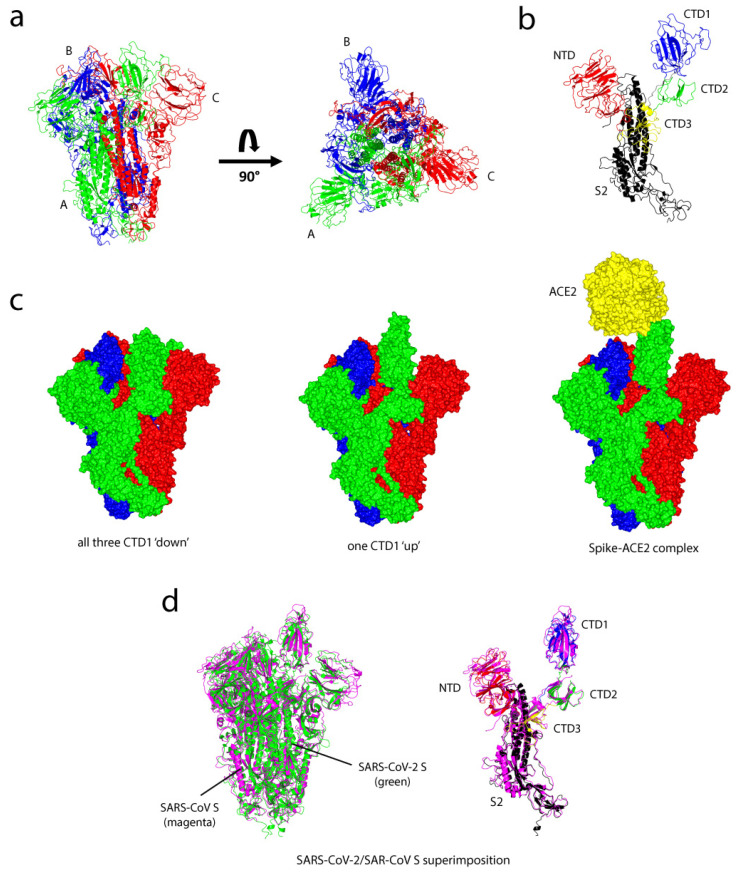
Structures of the severe acute respiratory syndrome coronavirus (SARS-CoV) spike glycoprotein and comparison with the severe acute respiratory syndrome coronavirus 2 (SARS-CoV-2) spike (S) glycoprotein. (**a**) Side and top views of the inactive prefusion structure of the SARS-CoV S glycoprotein, which adopts a ‘mushroom-like’, homotrimeric arrangement of S1/S2 subunits: chain A (green), chain B (blue) and chain C (red). (**b**) A single chain of the S glycoprotein which consists of S1 subunit: N-terminal domain (NTD) (red), the receptor-binding domain (RBD) of C-terminal domain CTD1 (blue), CTD2 (green) and CTD3 (yellow), and S2 subunit (black). (**c**) Three different conformational states of the SARS-CoV S glycoprotein: inactive S homotrimeric glycoprotein complex with all three CTD1 ‘down’ (PDB: 6ACC), active S glycoprotein with one CTD1 ‘up’ (PDB: 6ACD) and the spike-angiotensin-converting enzyme 2 (ACE2) complex (ACE2 shown in yellow) (PDB: 6ACG) [37]. (**d**) Superimpositions between the SARS-CoV-2 (PDB: 6VSB) [38] and SARS-CoV S glycoproteins. Left panel: SARS-CoV-2 (green), SARS-CoV (magenta). Right panel: chain A of the SARS-CoV-2 S glycoprotein (NTD is in red, CTD1 is in blue, CTD2 is in green, CTD3 is in yellow and S2 is in black) superimposition onto the SARS-CoV S glycoprotein (magenta) using SSM matching, as implemented in CCP4MG; root-mean-square deviation (RMSD): 1.97 Å over 884 Cα atoms.

The transmembrane spike (S) glycoprotein of SARS-CoV-2, like the S protein of SARS-CoV, facilitates coronavirus entry into host cells [24]. The S protein forms a homotrimeric complex protruding from the viral surface and consists of two functional subunits, S1 and S2, which are responsible for host cell receptor binding and the viral fusion to the host cellular membranes (Figure 1). The smaller S1 subunit consists of an N-terminal domain (NTD) and three C-terminal domains (CTD1–3), of which CTD1 forms the receptor-binding domain (RBD) and contributes to the stabilisation of the membrane-anchored S2 subunit (Figure 1b). The larger S2 subunit contains the machinery for viral fusion and comprises a hydrophobic fusion peptide (FP), heptad repeat 1 (HR1), central helix (CH), connector domain (CD), heptad repeat 2 (HR2), transmembrane domain (TM) and cytoplasmic tail (CT) [24,25,26,27,28]. The HR1 is vital for the S glycoprotein stability, maintaining the correct protein fold in the closed pre-fusion conformation [29]. In all coronaviruses, the S glycoprotein is cleaved by host proteases at the S1/S2 junction; cleavage has been suggested to activate the protein for host membrane fusion through irreversible conformational changes. There is a second cleavage site, S2′, located 130 residues from the N terminus of the S2 subunit which is highly conserved among coronaviruses. Cleavage at the S2’ site by host cell proteases is vital for successful infection by coronaviruses [30,31,32,33]. Unlike MERS-CoV, which uses the human entry receptor DPP4 [34], all SARS-related coronaviruses such as SARS-CoV and SARS-CoV-2 interact directly with the host cell receptor angiotensin-converting enzyme 2 (ACE2). ACE2 is a protease that is responsible for blood pressure and volume regulation; it is widely expressed on the cell membranes of the lung, heart, kidneys and gastrointestinal tract [35], although the lung is the main tissue for SARS-CoV-2 infection. Furthermore, host cell entry of SAR-CoV-2 depends on the serine protease TMPRSS2 for S protein priming and is essential for viral spread in the infected host [36].

Structures of three different conformational states of the homotrimeric SARS-CoV S glycoprotein in complex with the host cell receptor ACE2 have been determined by cryoelectron microscopy (cryo-EM) [37]. The structures consist of three S1/S2 heterodimers and bind the cellular receptor ACE2 to mediate viral fusion with cellular membranes through pre- to post-fusion conformational changes (Figure 1b). The inactive, ‘down’ conformational state is where the RBD of the S1 subunit is orientated 22.7° between the long axes of the RBD and the horizontal plane. This ‘down’ conformation corresponds to the receptor-inaccessible state (Figure 1c). Upon the binding to ACE2, the RBD of the trimeric S glycoprotein adopts a protruding ‘up’ conformation which then promotes the release of the S1 subunit and triggers a substantial structural rearrangement to fuse the viral membrane with the host cell membrane [37]. One CTD1 domain adopts an active ‘up’ conformation as a prerequisite for the binding of S glycoprotein to ACE2: the domain is important for the neutralisation by monoclonal antibodies (mAbs) and vaccine design [31].

The cryo-EM structure of the S glycoprotein of SARS-CoV-2 in the prefusion conformation has recently been determined [38]. The structure adopts the same homotrimeric arrangement of the S1/S2 subunits and shows a striking similarity with the homotrimeric S glycoprotein of SARS-CoV (Figure 1d). Unlike the RBD of the SARS-CoV, which is tightly packed against the NTD of the neighbouring chain, the RBD of the SARS-CoV-2 is angled closer to the central cavity of the trimer, suggesting slightly different conformations between the two S glycoproteins [38]. Despite this difference, the SARS-CoV-2 S glycoprotein monomer in the ‘up’ conformation is structurally similar to the S glycoprotein from SARS-CoV with a root-mean-square deviation (RMSD) of 1.97 Å (Figure 1d). Moreover, the SARS-CoV-2 S glycoprotein binds to ACE2 with high affinity (~15 nM): 10-fold higher than SARS-CoV S binding to ACE2 (150 nM) [38,39]. The high affinity of binding is a contributory factor to how the virus is spread easily among human populations.

## 4. RBD/ACE2 Complexes

**Figure 2:**
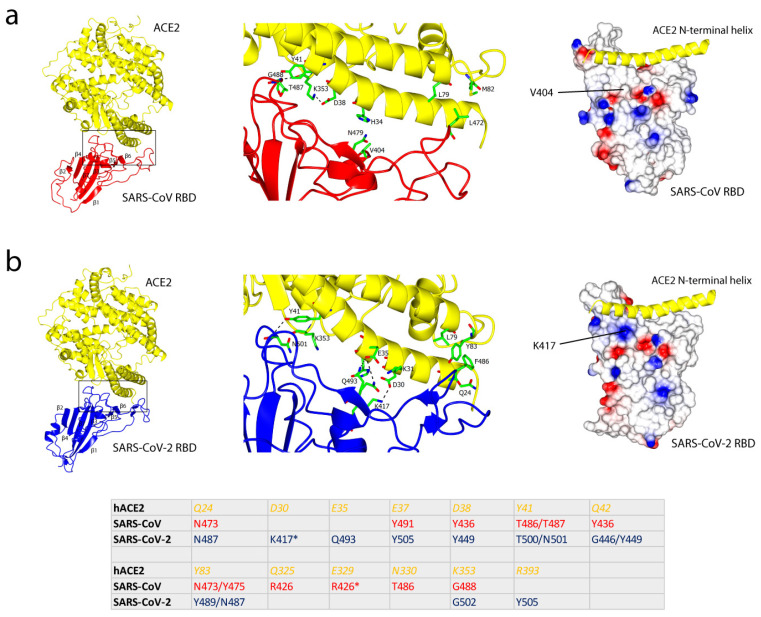
The SARS-CoV and SARS-CoV-2 RBD/ACE2 complexes. (**a**) Left: the overall structure of the SARS-CoV RBD (red) bound to ACE2 (yellow). The region contains a receptor-binding motif (RBM) which is highlighted with a box. Middle: L472 of the SARS-CoV RBD interacts with L79 and M82 of ACE2, N479 of the SARS-CoV RBD with H34 of ACE2, a hydrogen bond between G488 of the SARS-CoV RBD and K353 of ACE2. There is no interaction of the SARS-CoV RBD V404 (equivalent to K417 in the SARS-CoV-2 RBD) with any residues from ACE2. Right: the electrostatic potential map of the SARS-CoV RBD, which shows an inward concave structure with the N-terminal helix of ACE2, shown in yellow (PDB: 2AJF) [40]. (**b**) Left: the overall structure of the SARS-CoV-2 RBD (blue) bound to ACE2 (yellow). Note the similarity between the two RBDs: the core subdomain consists of 5 antiparallel β strands (β1—4 and β7) and is conserved. Middle: F486 of the SARS-CoV-2 RBD interacts with Q24, L79 and Y83 of ACE2, a hydrogen bond between N501 of the SARS-CoV-2 RBD and Y41 of ACE2, two hydrogen bonds between Q493 of the SARS-CoV-2 RBD and E35 of ACE2, a salt-bridge between K417 of the SARS-CoV-2 RBD and D30 of ACE2. Right: the electrostatic potential map of the SARS-CoV-2 RBD with the N-terminal helix of ACE2 (PDB: 6M0J) [41]. Bottom panel: the table summarises key amino acid residues which form hydrogen bonds and salt bridges between ACE2 and the RBD of SARS-CoV and SARS-CoV-2. There are 13 hydrogen bonds and 3 salt bridges between ACE2 and the SARS-CoV RBD, and 13 hydrogen bonds and 2 salt bridges between ACE2 and the SARS-CoV-2 RBD. Y436 of the SARS-CoV RBD forms two hydrogen bonds with D38 of ACE2. Residues that form salt bridges are labelled with an asterisk [40,41].

The crystal structure of SARS-CoV RBD bound to ACE2 (SARS-CoV RBD/ACE2 complex) has been determined to a resolution of 2.9 Å [40]. The structure shows an inward concave surface that is complementary to the ACE2 receptor tip, with 1,699 Å^2^ of buried surface between the two interfaces. The RBD of SARS-CoV consists of two subdomains: a highly conserved core and an external loop subdomain, which interacts with ACE2. The core subdomain is composed of five antiparallel β strands (β1—4 and β7), with a disulfide bond between the two β strands (formed by C366 and C419). The external subdomain mainly consists of loops, turns and two short β strands (β5 and β6), and forms a slight inward surface (Figure 2a). The overall structure of the SARS-CoV RBD serves as a cradle for the N-terminal helix of the peptidase ACE2. A total of 16 residues from the SARS-CoV RBD, which constitute most of the receptor-binding motif (RBM), are in contact with 20 residues of the ACE2 receptor, strengthened by 13 hydrogen bonds and 3 salt bridges between the two molecules (Figure 2) [41]. T487, which lies in a hydrophobic region at the RBD/ACE2 interface, appears to enhance human-to-human transmission of SARS-CoV. The methyl group of T487 lies in a hydrophobic region at the SARS-CoV RBD/ACE2 interface and is a key factor in determining transmissibility of the infection: a mutation has been observed to reduce the binding affinity by more than 20-fold [40].

The structure of the SARS-CoV-2 RBD bound to the cell receptor ACE2 has been recently determined [41] and, similar to the SARS-CoV RBD, the RBD of SARS-CoV-2 also has a core subdomain, formed from a five-stranded antiparallel β sheet, and an external loop subdomain, which serves a pocket for the N-terminal helix of ACE2 binding (Figure 2b). The overall ACE2-binding mode of the SARS-CoV-2 RBD is almost identical to that of the SARS-CoV RBD/ACE2 complex, in which the RBM is located in the external loop subdomain and mediates the binding. A buried surface of 1687 Å^2^ results from the cradling of the N-terminal helix of ACE2 by the inward concave surface of the SARS-CoV-2 RBD, and shows a high degree of similarity with the recognition mediated by SARS-CoV RBD. The overall structure of the SARS-CoV-2 RBD is similar to that of the SARS-CoV RBD, with an RMSD of 1.2 Å from an overlap of the structures. Compared to SARS-CoV RBD, a higher number of residues from SARS-CoV-2 RBD interact with ACE2 [41], which may explain its 10-fold higher binding affinity [38]. One, K417, lies outside of the RBM and forms a salt-bridge connection with D30 of ACE2. Its counterpart in the SARS-CoV RBD is Val, which shows no interaction with any residues in ACE2. K417 contributes to a positively charged patch on the SARS-CoV-2 RBD surface which connects to the negatively charged Asp for a stronger ACE2 binding [41].

## 5. SARS-COV-2 Variants

Viruses naturally accumulate mutations over time; since SARS-CoV-2 was first identified in China, thousands of mutations have been recorded [42]. The vast majority of these mutations have a little apparent impact on the viral infectivity but some give rise to lineages that are selected because they confer an advantage in transmission or carriage, and hence lead to a rapid rise in COVID-19 cases worldwide. Although the antibody-resistant SARS-CoV-2 variants are present at low frequencies in circulating SARS-CoV-2 populations, it is worth noting that these variants could limit the therapeutic use of vaccination, and achieving herd immunity would therefore be more challenging. Notable variants of SARS-CoV-2 that have spread include cluster 5, lineages B.1.1.7 (Alpha), B.1.351 (Beta), P.1 (B.1.1.28/Gamma), B.1.427/B.1.429 (Epsilon), B.1.526 (Iota) as well as B.1.617.2 (Delta). In this review, we discuss the structures of these variants and their interactions with neutralising antibodies (nAbs) and thus provide some insights into their transmissibility and neutralisation escape.

## 6. Cluster 5

**Figure 3:**
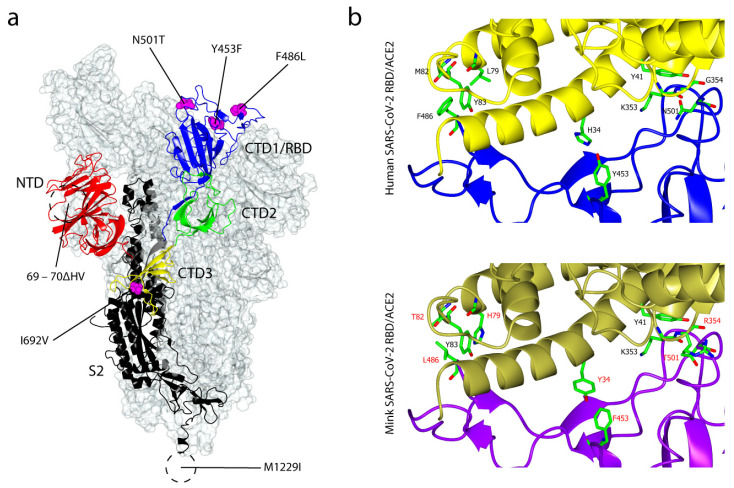
Mink-derived Cluster 5 of SARS-CoV-2 S glycoprotein mutations and their impact on the ACE2 interface. (**a**) The location of a deletion and five substitution mutations on the cryoelectron microscopy structure of the mink-derived SARS-CoV-2 S glycoprotein at 2.83 Å (PDB: 7LWM). Three mutations, Y453F, F486L and N501T, lie within the RBD (CTD1) of the SARS-CoV-2 S protein, the deletion mutation 69–70ΔHV is in the NTD, I692V is in the CTD3 and M1229I is in the hydrophobic region of the SARS-CoV-2 S glycoprotein. (**b**) A comparison between human SARS-CoV-2 RBD/ACE2 (top) and mink SARS-CoV-2 RBD/ACE2 (bottom). In humans, residues that are important in the ACE2 (yellow) binding are F486, Y453 and N501 within the SARS-CoV-2 RBD (blue). These residues are replaced by Leu, Phe and Thr, respectively in mink SARS-CoV-2 RBD (purple). H79, T82 and Y83 of the mink ACE2 (gold) interact with L486, Y34 interacts with F453, Y41, K353 and R354 interact with T501. As the mink SARS-CoV-2 RBD/ACE2 structure has not been determined, the human SARS-CoV-2 RBD/ACE2 structure (PDB: 6M0J) [41] was used for reference and modelling was performed using PyMol.

Cluster 5, also known as ΔFVI-spike, is a variant of SARS-CoV-2 which has largely disappeared from the current pandemic. It was first discovered in Denmark in early November 2020 and is believed to have been spread from minks to humans following a natural reverse-zoonotic transmission, from humans to minks which were reported earlier in two mink farms in the Netherlands [43,44]. As of 5 November 2020, there were 214 human COVID-19 cases infected with SARS-CoV-2 related to mink, all of which carried the mutation Y453F in the S glycoprotein. Of those, 12 cases were infected with a strain bearing four genetic mutations in the S glycoprotein: 69–70ΔHV, Y453F, I692V and M1229I [45], named Cluster 5 (Danish Serum Statens Institute). Additionally, two more substitution mutations were observed from the mink-related Dutch human cases: F486L and N501T [46]. These substitutions, however, were not found in the Danish cases. Y453F, F486L and N501T are important as they lie in the RBD of the SARS-CoV-2 S glycoprotein, and are predicted to be directly involved in the binding to ACE2. A recent molecular modelling study showed that these mutations increase the binding affinity, suggesting a potential adaptation of the SARS-CoV-2 S glycoprotein to the mink ACE2 [46]. Moreover, residues L486, F453 and T501 appear to provide a better fit to the interacting mink ACE2 residues (Figure 3). At positions 79 and 82, His and Thr replace Leu and Met, respectively in the mink ACE2, and interact with L486. At position 34, Tyr replaces His and interacts with F453. At position 354, Arg replaces Gly and interacts with T501. These differences suggest the viral selective adaptation to the new host, by optimizing its spike protein binding affinity to the mink ACE2 [46]. In addition, the Danish Y453F Cluster 5 variant binds the human ACE2 receptor with a four-fold increase in affinity compared to the original strain (3.85 nM vs 15.5 nM) [47]. Although it binds at an apparent higher affinity to the human ACE2, the Y453F variant does not affect the neutralising antibody (nAb) response in a vaccine mouse model. No differences in the inhibition potency of the two RBD variants—the Y453F and original strain—were reported, suggesting that the substitution mutation in Cluster 5 is a way to adapt and optimise receptor binding, rather than towards an immune evasion strategy [47]. It is worth noting the role of mink in the spread of SARS-CoV-2, as it provides evidence of the zoonotic animal-to-human, animal-to-animal and anthroponotic human-to-animal transmissions [44,48].

## 7. Lineage B.1.1.7 (Alpha Variant)

**Figure 4:**
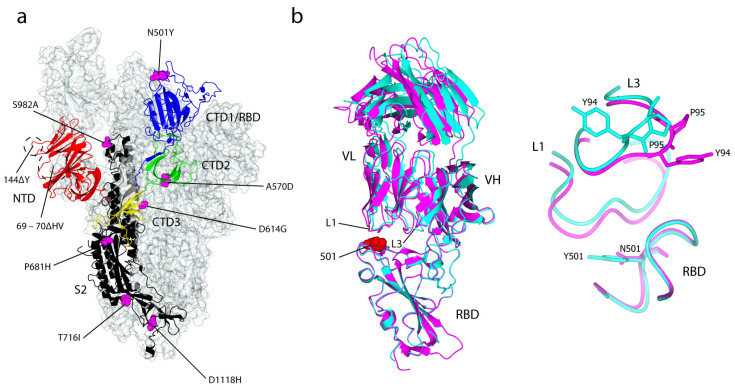
Location of B.1.17 variant mutations antibody recognition of the SARS-CoV-2 S glycoprotein. (**a**) The locations of seven substitutions and two deletion mutations within the UK B.1.1.7 SARS-CoV-2 S glycoprotein at 3.22 Å (PDB: 7LWU) [54]. Only one mutation: N501Y, lies within the RBD and two deletions: 69–70ΔHV and 144ΔY are within the NTD of the S protein. The D614G mutation presents in all B.1 lineages. (**b**) Left: the superimposition of the N501 RBD/Fab 269 complex (magenta; PDB: 7NEH) onto the Y501 RBD/Fab 269 complex (cyan; PDB: 7NEG) [52]. The location of residue 501 is shown, which in contact with the L1 and L3 loops of Fab 269. Right: an apparent displacement of the L1 loop and concomitant effect on the L3 loop, caused by the mutation of Asn to Tyr at position 501.

A SARS-CoV-2 variant of concern (VOC), lineage B.1.1.7, also referred to as VOC 202012/01, 20I/501Y.V1 or Alpha variant (WHO), was first reported on 14 December 2020 in the United Kingdom and has now been detected in over 160 countries [49]. It spread rapidly in the UK and became the dominant circulating variant, leading to travel restrictions and lockdowns throughout the country. As of 19 May 2021, there are almost 250,000 B.1.1.7 variant cases reported in the UK [50]. B.1.1.7 carries a constellation of genetic mutations, notably N501Y, which also presents in B.1.351 and B.1.1.28, in the S protein RBD (Figure 4a). N501 is involved in hydrophobic interactions with the side chains of residues Y41 and K353 of the ACE2 receptor; mutation from Asn to Tyr enhances these interactions. This mutation is predicted to affect the structural conformation of the RBD and thus the binding affinity for ACE2 [51,52,53]. It was reported that the B.1.17 variant binds to the ACE2 receptor with a 5-fold higher affinity than the S-D614G variant [54]. Furthermore, B.1.1.7 RBD bound ACE2 with 2-times greater affinity than the wild-type S RBD [55]. The N501Y mutation also can be found in two other unrelated variants: B.1.351 (detected in South Africa) and P.1 (originated from Manaus, Brazil). This mutation, like other mutations that lie within the RBD of the SARS-CoV-2 S glycoprotein, may escape antibody neutralisation by disrupting antibody binding. This phenomenon is particularly pronounced for mAb 278, which attained a maximum of 78% neutralisation and mAb 269, where neutralisation was almost completely abolished. Other nAbs, however, were minimally affected by these mutations and still retained potent neutralising activity [52]. Only two (910-30 and S309) out of twelve RBD-directed mAbs showed a reduction in neutralisation activities against B.1.1.7, in which the significant decrease activity of 910-30 is mediated by the N501Y mutation. Interestingly, all six NTD-directed mAbs showed major reductions in neutralisation against the variant, with antibodies 5-24, 4-8 and 4A8 exhibiting a total loss of activity. In addition, the activities of 2-17, 4-19 and 5-7 were variably impaired. These reductions in the neutralisation activities of the NTD-directed mAbs are largely attributable to the 144ΔY deletion mutation, which falls within the NTD antigenic supersite [56].

In addition, a recent study using sera from convalescent patients and vaccine recipients showed a modest reduction in the neutralisation against the B.1.1.7 variant, although it was not completely abolished [52]. Sera obtained from the individuals who have received the AstraZeneca vaccine showed 2.1- to 2.5-fold reductions in neutralisation, whereas, for sera obtained from the individuals with the Pfizer-BioNTech vaccine BNT162b2, there was a 3.3-fold reduction in the neutralisation against the B.1.1.7 variant. Moreover, a moderate reduction (about 2-fold) in neutralisation was also observed using serum samples from recipients of the Moderna mRNA-1273 and the Novavax NVX-CoV2373 vaccines [57]. Furthermore, the Oxford-AstraZeneca AZD1222 vaccine showed efficacy against the B.1.1.7 variant, although a reduction in neutralisation activity was observed against the variant compared with other variants. Vaccine efficacy against the symptomatic infection caused by the B.1.1.7 variant was 70.4%, compared to 81.5% efficacy against the other variants [58]. These findings indicate that the B.1.1.7 variant indeed does not totally escape neutralisation and, for now, it is not a major concern for the current deployment of COVID-19 vaccines. However on 2 February 2021, Public Health England has reported that a number of B.1.1.7 cases were detected with the E484K mutation [59], also present in the Brazil and South Africa variants. The E484K mutation has been attributed to the reduced neutralisation of the P.1 and B.1.351 variants [56,60]. These observations have raised concerns about vaccine effectiveness and further investigations are required to determine what effects that the new E484K mutation may have on the B.1.1.7 variant.

**Figure 5:**
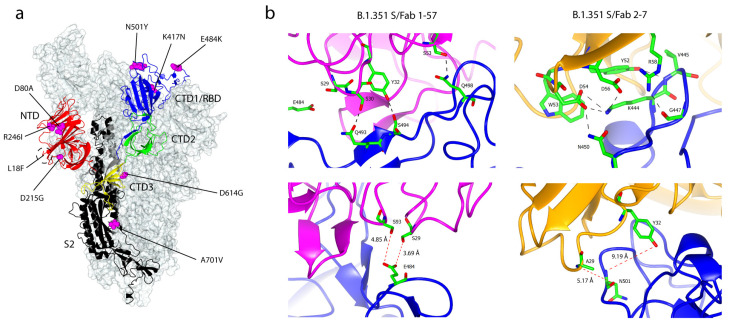
Mutations within the B.1.351 variant of SARS-CoV-2 S glycoprotein and recognition by Fab 1-57 and Fab 2-7. (**a**) The locations of nine substitution mutations on the structure of the South African B.1.351 SARS-CoV-2 S glycoprotein at 3.32 Å (PDB: 7LYN) [54]. Three mutations- K417N, E484K and N501Y- lie within the RBD. Four mutations- L18F, D80A, D215G and R246I- are in the NTD. There is no deletion mutation in the variant. (**b**) Left: recognition of CDR-L1 and CDR-L2 of the Fab 1-57 (magenta) on the RBD (blue). Residues that are involved in binding are labelled. Hydrogen bonds are represented as black dash lines (less than 3.2 Å). E484 did not interact with any residues as it is too distant from S29, S93 and V100 (not shown). The distances of E484 to S29 and S93 are 3.69 Å and 4.85 Å, respectively (PDB: 7LS9) [61]. Right: recognition of CDR H2 from Fab 2-7 (orange) of the RBD (blue). Residues that are involved in binding are labelled. Hydrogen bonds are represented as black dash lines. N501 did not interact with any residues on Fab 2-7. The distances of N501 to A29 and Y32 are 5.17 Å and 9.19 Å, respectively—too distant for the formation of hydrogen bonds (PDB: 7LSS) [61].

There is evidence that the N501Y mutation causes changes to the binding conformation of the RBD. Based on the structure of the SARS-CoV-2 S RBD in complex with the antigen-binding fragment (Fab) 269, and in comparison with the structure of the B.1.1.7 RBD/Fab 269 complex, the N501Y mutation introduces a small displacement of the L1 loop of Fab 269 (V_L_ chain) (Figure 4b). The mutation also induces an associated major displacement of the neighbouring L3 loop: Y94 position is switched to facing inwards, followed by a small twist of P35 [52]. Interestingly, the neutralisation potency of two potent RBD-directed antibodies, 1-57 and 2-7, was unaffected by the B.1.1.7 variant [61]. The cryo-EM structures of these potent 1-57 and 2-7 antibodies, in complex with the S glycoprotein, have been determined (Figure 5b). Both 1-57 and 2-7 nAbs target epitope residues outside evolutionary pressure ‘hotspots’ on the ACE2 receptor binding, suggesting that they could be used for therapeutic purposes [61]. Several other mutations in the S protein include a deletion at positions 69 and 70 (69–70ΔHV), which evolved spontaneously in at least 28 other SARS-CoV-2 lineages. This mutation is hypothesised to increase viral transmissibility and infectivity, and often occurs simultaneously with N439K, Y453F and N501Y mutations in other variants [62]. Moreover, it has been estimated that the B.1.1.7 variant is 75% more transmissible compared with the wild type strain [63]. A recent transmissibility assessment reported that the B.1.1.7 variant has a 50% to 100% higher reproduction number than non-B.1.1.7 lineages [64]. The variant was previously estimated to be 30% to 60% more infectious than strains encountered in the first wave of the pandemic [65].

## 8. Lineage B.1.351 (Beta Variant)

On 18 December 2020, South Africa reported a SARS-CoV-2 variant with several mutations that affect the S glycoprotein. The new South African lineage (20H/501Y.V2 or B.1.351 or Beta variant) emerged after the first pandemic wave, in Nelson Mandela Bay, on the coast of Eastern Cape Province. The variant is characterised by eight lineage-defining mutations in the S protein, including three substitutions of the important residues within the RBD- K417N, E484K and N501Y- which affect the binding affinity to ACE2 [66]. These mutations also arose independently in the Brazilian B.1.1.28 variant. Four other mutations, L18F, D80A, D215G and R246I, are located in the NTD but only one (A701V) is in the S2 subunit. Like many other B.1 lineages, the variant also has the B.1-defining mutation, D614G, in the CTD3 (Figure 5a). Like the B.1.1.7 variant, the B.1.351 binding affinity to ACE2 increased significantly relative to the S-D614G variant: the increase is attributed to the N501Y mutation in both variants [54]. The B.1.351 RBD bound ACE2 with 4.62-times greater affinity than the wild-type S RBD [55]. The addition of two more substitution mutations in the RBD contributes to the higher binding affinity. Using ELISA and surface plasmon resonance (SPR), the binding of the B.1.351 variant to the neutralising NTD-directed antibody DH1050.1 was dramatically reduced, due to multiple mutations in the NTD. The RBD-directed antibodies DH1041 and DH1043, similarly, bound with lower affinity to the B.1.351 variant, suggestive of an allosteric effect of the NTD mutations on the B.1.351 binding to DH1041 and DH1043. The binding of the B.1.351 variant to the cross-reactive RBD-directed antibody DH1047 and the fusion peptide-directed antibody DH1058 remained unaffected [54]. In addition, the binding affinities of the RBD-directed nAbs 1-57 and 2-7 were also unimpaired by the B.1.351 lineage [61], despite having three mutations in the RBD: this observation suggests a possible therapeutic application in the neutralisation of the variant, like that of B.1.1.7. Structural analysis of the Fab 1-57 binding to the S glycoprotein revealed that the antibody accommodates the E484K mutation in a hydrophilic pocket and E484 did not significantly interact with 1-57 (Figure 5b). Structural modelling of E484K showed that the Lys substitution was compatible with binding to the Fab 1-57 as it lies outside of the binding footprint [61].

The B.1.351 variant is resistant to a group of mAbs that bind the RBM of the RBD, including three antibodies that are approved for emergency use in the treatment of COVID-19 infection. The neutralisation activities of the RBM-directed antibodies 2-15, LY-CoV555 (bamlanivimab), C121 and REGN10933 (casirivimab) were completely abolished. The activity of the 910-30 antibody, which is directed against the inner side of the RBD, was also markedly impaired [56]. The complete impairment of neutralisation activities of 2-15, LY-CoV555 and C121 against the B.1.351 variant is attributed to the E484K mutation, whereas the K417N substitution contributes to the marked reduction in the neutralisation activity of 910-30. The complete loss of activity of REGN10933, on the other hand, is attributed to both the K417N and E484K mutations [67]. The activities of three out of six NTD-directed antibodies (5-24, 4-8 and 4A8), all of which target the antigenic supersite in the NTD, were completely abolished, whereas 2-17, 4-19 and 5-7 antibodies showed variable impairment against B.1.351. The resistance of B.1.351 against these NTD-directed antibodies is largely caused by the R246I substitution mutation, which lies within the NTD-antibody binding supersite. In addition, the neutralisation activities of sera obtained from individuals vaccinated with the Moderna mRNA-1273 and the Pfizer BNT162b2 vaccines were reduced 12.4- and 10.3-fold respectively. Resistance is thought to be mediated by the E484K mutation, situated in an immunodominant epitope of the RBM [56]. Moreover, a study using pseudoparticles with immortalised cell lines showed that host cell entry driven by the S proteins of the B.1.351 variant was less susceptible to inhibition by sera collected from individuals vaccinated with the Pfizer BNT162b2, compared to the wild-type S protein [68]. However, a recent study showed that the Oxford-AstraZeneca AZD1222 vaccine gives protection to hamsters against both the B.1.351 and B.1.1.7 variants, although a 9.5-fold in reduction of nAb was observed against B.1.351 compared to B.1.1.7 [69]. Taken together with interim results from a Novavax vaccine trial in South Africa, in which only 60.1% of efficacy was reported against the B.1.351 variant, these results raise concern about protection against arising variants from these first-generation vaccines. When an additional 240 HIV-infected individuals were included, protection from the Novavax vaccine dropped to 49.4% [70].

## 9. Lineage P.1 (B.1.1.28/Gamma Variant)

**Figure 6:**
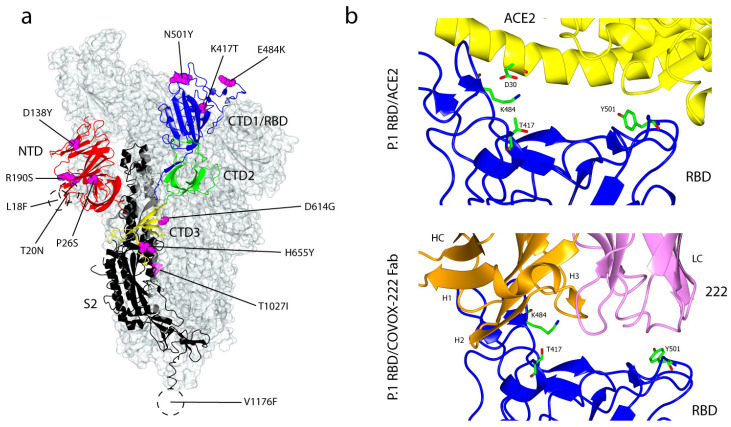
Mutations within the P.1 (B.1.1.28) variant of SARS-CoV-2 S glycoprotein and its interactions with ACE2 and COVOX-222 Fab. (**a**) The locations of ten substitution mutations within the Brazilian B.1.1.28 SARS-CoV-2 S glycoprotein at 3.00 Å (PDB: 7LWW) [54]. Similar to the South African B.1.351 lineage, there are three mutations in the RBD: K417T, E484K and N501Y and no deletion mutations in the variant. V1176F is in the hydrophobic region of the SARS-CoV-2 S glycoprotein. (**b**) Top panel: the crystal structure of P.1 RBD (blue) in complex with ACE2 (yellow). T417 did not form a salt bridge with D30 of ACE2 (PDB: 7NXC) [60]. Bottom panel: the crystal structure of P.1 RBD (blue) in complex with COVOX-222 Fab (HC is in orange and LC is in pink). The interactions between the two molecules are mostly mediated by CDR-H1 and CDR-H2, which did not interact with T417 (PDB: 7NXB) [60].

Lineage P.1, also known as B.1.1.28 or 20J/501Y.V3 or Gamma variant (WHO), was first detected by the National Institute of Infectious Disease, Japan in four visitors arriving from Brazil on 6 January 2021. The variant caused a widespread infection in Manaus, where it was identified in 42% of samples collected in December 2020 [71]. The sharp increase in the total COVID-19 infections, followed by an increase in the number of hospital admissions observed in Manaus, indicate the high transmissibility of the variant [72,73]. It has now been detected in more than 200 cases in Canada [74], Italy [75], the USA [76] and Belgium, and the total world infection has now reached 2,300 cases (as of 5 April 2021). Information on the transmissibility of the P.1 variant, however, is scant; it shares several mutations that were acquired independently with the B.1.351 variant- K417T, E484K and N501Y- all of which in the RBD of the S glycoprotein (Figure 6a). In P.1, Thr replaces Lys at residue 417, whereas Asn replaces Lys in the B.1.351 variant. These mutations have been shown to increase the binding affinity of the B.1.351 S glycoprotein to ACE2 and the N501Y mutation is suggested to largely contribute to the increase in the binding affinity [54,66]. In a recent study, the equilibrium binding affinity constant of P.1 to ACE2 is 4.8 nM, similar to the B.1.351/ACE2 binding, which was previously recorded at 4.0 nM. The binding affinity is largely attributed to the three RBD mutations, in which the mode of the interaction to ACE2 is essentially identical for P.1 and B.1.351. The crystal structure of the P.1 RBD/ACE2 complex has been determined recently (Figure 6b). In the wild type SARS-CoV-2, K417 forms a salt bridge with D30 of the ACE2 receptor (Figure 2b) but the Thr of P.1 forms no equivalent contact. The replacement of the negatively-charged Glu with a positively-charged Lys at residue 484, nevertheless, contributes to a marked change in overall charge which improves the electrostatic complementarity, leading to higher affinity binding. Furthermore, the substitution of the Asn sidechain at residue 501 with Tyr promotes favourable ring stacking interactions, consistent with stronger affinity [60]. Other mutations which define the P.1 variant are five substitution mutations (L18F, T20N, P26S, D138Y and R190S) in the NTD and three substitutions (D655Y, T1027I and V1176F) in the S2 subunit.

Although P.1 has more mutations in the S protein compared to the B.1.351 variant, surprisingly, it is significantly less resistant to the neutralisation by nAbs, whether acquired naturally or induced by vaccinations. Nevertheless, many RBD-directed mAbs showed reduced or complete loss of neutralisation against P.1; for example, neutralisation of P.1 by LY-CoV555 and LY-CoV16 nAbs was almost completely reduced. REGN10933 and AZD8895 caused significant and modest decreases in neutralisation, respectively. The neutralisation escape from REGN10933 and LY-CoV555 is severely compromised by the substitution E484K, in which both antibodies form strong interactions with residues 484-486. LY-CoV016 antibody, on the other hand, is affected by changes at residues 417 and 501. In addition, the neutralisation of the NTD-directed mAb159 was reduced 133-fold on P.1 compared to the Victoria strain due to the epitope disruptions by the five NTD mutations of P.1. Interestingly, the neutralisation of the VH3-53 222 mAb was unaffected by the changes in P.1 and no apparent reduction was observed in the binding affinity of 222 to both P.1 and Victoria RBD. The crystal structures of 222 in complex with the P.1 RBD suggested that most of the binding energy comes from CDR-H1 and CDR-H2, which do not interact with the residue 417 in the RBD (Figure 6b) and hence, the substitution from Lys to either Asn or Thr does not affect binding affinity or neutralisation. Furthermore, neutralisation of P.1 by the Pfizer BNT162b2 and the Oxford-AstraZeneca AZD1222 vaccines showed a reduction of 2.6-fold and 2.9-fold, respectively relative to the Victoria strain, considerably better than the neutralisation of the B.1.351 variant, where 7.6-fold and 9.0-fold of reductions were observed, respectively [60].

In a separate study, P.1 is shown to be more resistant to neutralisation by nAbs, convalescent plasma and vaccine sera [77]. Three mAbs with Emergency Use Authorization (EUA) were shown to have markedly or completely abolished neutralisation against P.1: REGN10933 (casirivimab), LY-CoV555 (bamlanivimab) and CB6 (etesevimab), whereas only REGN10987 (imdevimab) retained its activity against P.1. The other two potent mAbs that targeted the RBM, 2-15 and C121, showed complete loss of neutralising activities against P.1, while six mAbs targeted the inner side or outer side of the RBD retained their neutralisation capacities. Furthermore, P.1 was resistant to four NTD-directed nAbs: 2-17, 4-18, 4-19, and 5-7. Two other NTD Abs, 5-24 and 4-8, retained their neutralisation capabilities against the P.1 variant, despite the total loss of activity against B.1.351 [56]. The P26S mutation partially contributes to a loss in neutralising activity of 4-18, while the loss of activity of 2-17 and 4-19 is attributed to mutations L18F, T20N and D138Y. The L18F, T20N, D138Y and R190S mutations altogether contribute to the loss of activity of 5-7. Interestingly, unlike B.1.351, the loss of neutralisation by vaccine sera obtained from individuals who received the Pfizer BNT162b2 and the Moderna mRNA-1273 vaccines against P.1 was modest (2.2-fold and 2.8-fold, respectively) [77].

## 10. Other Notable Lineages

Since the emergence of the SARS-CoV-2 virus, many variants have been reported; some are particularly important due to their increased virulence and transmissibility as well as their abilities to evade the immune response. Lineage B.1.427/B.1.429, also known as CAL.20C or Epsilon variant (WHO), is a novel VOC that was originally detected in California in July 2020. This variant is now spreading in 30 countries, particularly the United States, although the number of cases remains relatively low [78]. The variant is defined by five distinct mutations: I4205V and D1183Y in the ORF1ab-gene, and S13I, W152C and L452R in the S glycoprotein [79]. The L452R mutation is of particular concern, as it is within the RBD and has been shown to be resistant to some nAbs [80]. The variant exhibits an estimated 18.6 to 24% increase in viral transmissibility relative to non-B.1.427/B.1.429 strains [81]. The neutralising activities of sera against the variant obtained from convalescent patients and vaccinated individuals (with either the Pfizer BNT16b2 or Moderna mRNA-1273 vaccines), were reduced by 6.7- and 2.0-fold. Similarly, 2.8 and 4.0-fold reductions in neutralisation by sera collected from individuals vaccinated with the Moderna and Pfizer vaccines [78]. Likewise, plasma obtained from nine convalescent patients showed a 4.9-fold reduction in neutralisation against B.1.427/B.1.429, compared to the wild-type variant. Furthermore, mutations in the B.1.427/B.1.429 S glycoprotein have been attributed to reduced sensitivity to the RBD- and NTD-directed nAbs. The neutralising activity of the RBD-directed CT-P59 (regdanvimab) antibody against the B.1.427/B.1.429 variant was significantly reduced, whereas LY-CoV555 (bamlanivimab) completely lost its neutralisation due to the location of L452R in its epitope. Moreover, all ten NTD-directed nAbs showed a complete reduction in neutralisation, as a result of the S13I and W152C mutations, which are located in the S glycoprotein NTD. The W152C mutation forms a disulfide bond with the neighbouring C136 and disrupts the overall arrangement of the NTD antigenic supersite which is recognised by the nAbs [78].

Since December 2020, there has been an increase in the number of SARS-CoV-2 genomes that contained the E484K substitution mutation in the S glycoprotein in the US. This led to several tests for isolates from COVID-19 patients for B.1.351, P.1 and other variants which harbour the E484K mutation. A majority of isolates obtained from screening in New York City containing the mutation E484K fell within a novel lineage, B.1.526 (Iota variant, WHO). This newly discovered B.1.526 variant has a set of common mutations in the S glycoprotein: L5F, T95I, D253G, E484K, D614G and A701V [82,83]. D253 lies in the antigenic supersite loop of the NTD and has been identified as one of the important residues that is in contact with most of the NTD-directed nAbs [84]. This mutation may play an important role in modifying the neutralisation epitope and hence may contribute to the immune escape of the B.1.526 variant. Likewise, the E484K mutation, which is situated at the RBD interface with the receptor ACE2, may also play an important role in the neutralisation escape of B.1.526, as for B.1.351 and P.1 [56,60]. However, sera obtained from convalescent patients and vaccinated individuals retained neutralisation against the B.1.526 variant, although with a modest reduction (3.8-fold and 3.4-fold reduction by convalescent and vaccinated sera, respectively) was observed as compared to the D614G strain [85]. Neutralisation against B.1.526 by the REGN10987 nAb, which binds to the side of the RBD, was robust and retained at full capacity but REGN10933, which binds to the top face of the RBD, lost 12-fold of its neutralisation capacity. The neutralisation of the combined REGN10933/REGN10987 antibody cocktail, however, remained high and robust [85].

Lineage B.1.617.2, also known as Delta variant (WHO) is from lineage B.1.617, which was first detected in the state of Maharashtra, India in late 2020 and has been associated with the second wave of COVID-19 infections in the country. It has now spread to 62 countries, including the UK, where it is currently the dominant variant, replacing the lineage B.1.1.7 (Alpha variant) [49,86]. This variant carries 10 mutations in the S glycoprotein, four of them are of particular concern: L452R, T478K, D614G and P681R [87], which may associate with high viral transmissibility and increase in neutralisation escape. Unlike its parent B.1.617 variant, the E484Q mutation is not present in the B.1.617.2 genome. The L452R mutation, which also presents in the lineage B.1.427/B.1.429 (Epsilon variant) has been shown to be associated with increased transmissibility and resistance to some nAbs [80,81]. A recent study showed that there was a reduction of 5.8-fold in neutralising activity against B.1.617.2 relative to wild-type SARS-CoV-2, significantly more reduced than against B.1.1.7. Although there was a significant reduction in neutralisation against B.1.617.2, the data published suggested that most individuals which received a complete two-dose BNT162b2 vaccine would be protected against the variant and associated disease [88]. In addition, another study using a panel of 20 sera collected from BTN162b2-immunised individuals showed that the neutralisation against B.1.617.2 was modestly reduced relative to the wild-type [89]. A negative case-control study conducted in England reported that the effectiveness of two doses of the Pfizer/BioNTech vaccine was modestly reduced from 93.4% against B.1.1.7 to 87.9% against B.1.617.2. The AstraZeneca vaccine, however, showed significantly more reduction in effectiveness against the variant; 59.8% effective against B.1.617.2 compared to 66.1% against B.1.1.7 [90].

## 11. Transmissibility and Immune Evasion of SARS-COV-2

Coronaviruses have caused several pneumonic pandemics in humans in the 21st century: SARS-CoV, MERS-CoV and SARS-CoV-2. In 2002, SARS-CoV emerged in Guangdong, China and infected more than 8,000 people with a total of 774 deaths in five continents [91]. Ten years later in mid-2012, a new coronavirus variant, MERS-CoV emerged in the Arabian Peninsula and spread to 27 countries, infecting about 2500 people and claiming 858 lives [92]. Less than ten years later, a new variant of coronavirus was discovered in Wuhan, China in December 2019. The new variant, SARS-CoV-2 is associated with the ongoing outbreak of atypical pneumonia that has so far, as of May 2021, affected more than 168 million and killed more than 3.5 million people in just over a year, globally [93]. Although SARS-CoV-2 appears to be less virulent than SARS-CoV or MERS-CoV, its rate of transmission is much higher. Various modes of transmission of SARS-CoV-2 have been proposed, including direct or indirect contacts, short-range respiratory droplets, long-range airborne transmission of aerosols, surface contamination and even faecal-oral route of transmission [94,95]. Although there has been much debate on the SARS-CoV-2 transmission routes between humans, the plausibility of it being an opportunistic airborne infection is unquestionable.

Infected individuals often present with respiratory-like illnesses that develop to severe pneumonia, suggesting that the lung is the primary tropism of SARS-CoV-2. The presence of the abundant cell receptor ACE2, particularly on the lung surface, serves as the entry point into host cells for both SARS-related coronaviruses. SARS-CoV-2 entry into host cells is mediated by its S glycoprotein. Upon binding, the S protein undergoes major structural conformational changes and the RBD dissociates to allow viral fusion into the cells, where fusion is mediated by its S2 subunit [24]. Thus, the transmissibility of the virus depends largely on its recognition and binding to the ACE2 receptor. As the pandemic has grown, the virus mutated to adapt to different cell receptors as well as to evade host immune response. Most mutations are neutral or detrimental to virus fitness, while some may provide selective advantages, such as increased infectivity, transmissibility and reduced effectiveness of the immune response. There have been thousands of mutations recorded so far in SARS-CoV-2, with D614G being the most common mutation: it is found in a high number of isolates worldwide and has been suggested to increase replication and human-to-human transmission [96,97,98]. D614G enhances viral replication by increasing the stability of virions in human lung epithelial cells and airway tissues [99]. Furthermore, the introduction of the D614N mutation into the S protein preparation resulted in a striking improvement in thermal stability [29]. Although it has been associated with high upper respiratory tract viral loads and increased transmission, the D614G mutation does not contribute to COVID-19 disease severity, suggesting that both infectivity and transmissibility do not correlate with the severity of disease [96,100]. Although it is important to address how the mutation may affect the severity of COVID-19, the impact of such an effect is challenging to predict: it remains difficult to assess precisely to what extent the mutation may confer disease severity, especially given the multiple factors that contribute to patient severity, such as age and underlying medical conditions. The D614G mutation was uncommon before March 2020 but, as the pandemic spread and by June 2020, it accounted for more than 74% of all published sequences [101]. Nonetheless, the mutation is unlikely to reduce the neutralisation by nAbs and the efficacy of vaccines in protecting against COVID-19 [99]. However, new mutations within SARS-CoV-2 can acquire resistance to neutralisation and enhanced transmissibility, which is concerning.

**Figure 7:**
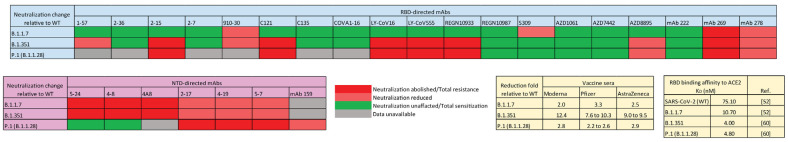
Neutralisation by RBD- and NTD-directed mAbs and vaccine anti-sera against B.1.1.7, B.1.351 and P.1 (B.1.1.28), relative to the wild-type virus. Neutralisation results, compiled from different studies which used several neutralisation assays: focus reduction neutralisation assay, where the reduction in the number of the infected foci is compared to a no antibody negative control well, was used in the neutralisation studies of B.1.1.7 [52] and P.1 [60], whereas end-point dilution neutralisation assay, where percentage neutralisation at a given sample dilution or mAb concentration is measured, was used in the neutralisation studies of B.1.1.7/B.1.351 [56] and P.1 [77]. The B.1.351 and P.1 variants potentially reduced the neutralisation of both RBD- and NTD-directed mAbs, while the B.1.1.7 variant showed reductions particularly in the neutralisation of the NTD-directed mAbs compared to the wild-type SARS-CoV-2. Vaccine sera used in these studies were obtained at 14 and 28 days following the second dose of the AstraZeneca AZD1222 vaccine [52,60] and 4-14 days [60] or 7-17 days [52,56] following the second dose of the Pfizer BNT162b2 vaccine. For the Moderna mRNA-1273 vaccine, the serum was collected 15 days following the second dose of the vaccine [56,77]. Although there were marked reductions in the neutralisation of vaccine sera, they remain robust, protecting these variants by the currently deployed vaccines. Bottom right: the table summarises the binding affinity (*K_D_*) of RBD of different SARS-CoV-2 variants to ACE2 using bio-layer interferometry [52,60].

Mutations of the important residues in the RBD of the S glycoprotein which affect binding to ACE2 (Figure 7), thus contribute to higher affinity and can lead to an increase in infectivity and transmissibility of the virus. When a virus switches host species, it adapts to the new ACE2 host receptor by increasing its mutation rate. Such naturally occurring RBD mutations might also be expected after a zoonotic or an anthroponotic event. For instance, a Y453F RBD mutation occurred after the anthroponotic human to mink transmission and the subsequent zoonotic mink to human transmission [46]. The Danish Cluster 5 variant, which contains the Y453F mutation in its RBD of the S glycoprotein, binds the human ACE2 cell receptor at a 4-fold higher affinity compared to the original strain [47]. This suggests that this mink-derived RBD substitution improves the binding of the S protein to the human ACE2 receptor, an indication of SARS-CoV-2 adaption to different species. Furthermore, in a mouse-derived SARS-CoV-2 strain, Q493K, Q498Y and P499T mutations are observed in its RBD [102], while tiger-adapted SARS-CoV-2 sequences contain an F456Y mutation and lion-linked SARS-CoV-2 contains a Y505H mutation in its RBD [103]. All of these residues have been suggested to interact with ACE2 and these substitutions may improve the binding affinity.

Mutations induce alterations in the protein structures of the virus, changing the antigenic properties of the strain, and can lead to reduced effectiveness of an immune response against the original strain. SARS-CoV-2 lineages B.1.1.7, B.1.351 and P.1 (B.1.1.28) are not only raising concerns because of their enhanced transmissibility but also because of their capabilities to reduce neutralisation by mAb therapies and vaccinations (Figure 7), associated with their multiple mutations in the S glycoprotein [54,56,60,77]. While the B.1.351 and P.1 variants potentially reduced the binding and neutralisation of both RBD- and NTD-directed antibodies, the B.1.1.7 variant, however, showed reductions in the binding and neutralisation of the NTD-directed antibodies. The acquired resistance of B.1.351 and P.1 to the RBD-directed nAbs is largely due to multiple mutations in their RBD, in which both of the variants share similar mutations: K417N/T, E484K and N501Y, located within the footprints of antibody binding. The impairment of antibody binding and thus the loss of neutralisation is attributed largely to mutations to residues 417 and 484. For example, the K417N mutation has been suggested to contribute to the marked reduction in the neutralising activity of 910-30 and the complete loss of neutralisation by REGN10933 against B.1.351. Conversely, the complete loss of activities of 2-15, LY-CoV555 and C121 were attributed to the E484K mutation. Likewise, E484K also has been shown to contribute to the significant reductions in the neutralisation of P.1 by REGN10933 and LY-CoV555: both antibodies form strong interactions with residues 484-486 [56,60,67]. The N501Y substitution has been attributed to the increase in ACE2 binding. This mutation is shared by the B.1.17, B.1.351 as well as P.1 variants, all of which have higher binding affinity to ACE2, compared to the wild type SARS-CoV-2 [52,54,60]. The 501 is primarily involved in hydrophobic interactions with the side chains of residues Y41 and K353 of the ACE2 receptor (Figure 2b). The aromatic structure of Tyr not only allows for ring stacking interactions which contribute to the higher binding affinity but is closer to the interacting residues on ACE2, contributing to stronger interactions. Two deletion mutations- 69–70ΔHV and 144ΔY- lie within the antibody binding footprints on the NTD of the S glycoprotein, disrupt the epitope residues and thus confer resistance to B.1.1.7 against the NTD-directed nAbs such as 2-17, 4-8, 4-19, 5-7, 5-24 and 4A8, all of which showed marked reductions in neutralisation against the variant. The 69–70ΔHV mutation has been shown to increase SARS-CoV-2 infectivity, is present in at least 28 different lineages and often coincides with the N439K, Y453F and N501Y mutations. The increase in infectivity is associated with higher spike cleavage and incorporation into virions during viral replication [62]. Moreover, the B.1.351 variant has four substitution mutations in its NTD and also showed significant impairment in its neutralisation by these NTD-directed antibodies. The resistance of B.1.351 is largely conferred by the R246I mutation, which lies within its NTD supersite [56]. Similarly, the P.1 variant, which has five substitution mutations in its NTD, is resistant to 2-17, 4-18, 4-19 and 5-7 NTD-directed antibodies. The P26S mutation solely contributes to the P.1 resistance against 4-18, while L18F, T20N and D138Y confer resistance against 2-17 and 4-19 antibodies. L18F, T20N, D138Y and R190S altogether contribute to the loss of neutralising activity of 5-7 [77].

Mutations in the S protein, which we discuss herein, do not only enhance viral fitness by improving binding affinity to the ACE2 receptor but also provide resistance against the immune response, acquired naturally or induced by vaccination. RBM, the ACE2-interacting surface of RBD, consists of a small patch of about 70 amino acids and is under constant selection pressure, shaping its adaptation and evolution. Many of these residues which form contact interfaces with ACE2 overlap antibody binding epitopes and, therefore, are constrained by mutation, thus affecting binding. It is imperative to gather comprehensive information concerning how mutations may affect the evolution of SARS-CoV-2 and thus would guide the development of vaccines and other therapeutics for protection against the ongoing COVID-19 pandemic.

## Data Availability

All data relevant to this review is included in the text and references.
